# Study on the Relationship between Manganese Concentrations in Rural Drinking Water and Incidence and Mortality Caused by Cancer in Huai'an City

**DOI:** 10.1155/2014/645056

**Published:** 2014-11-03

**Authors:** Qin Zhang, Enchun Pan, Linfei Liu, Wei Hu, Yuan He, Qiujin Xu, Cunzhen Liang

**Affiliations:** ^1^Huai'an City Center for Disease Prevention and Control, Huai'an, Jiangsu 223001, China; ^2^The Department of No-Communicable Diseases Control and Prevention, Huai'an City Center for Disease Prevention and Control, 118 Huaihai North Road, Huai'an, Jiangsu 22300, China; ^3^China Research Academy of Environmental Science, Beijing 100012, China; ^4^Department of Environmental Engineering, Beijing Petrochemical College, Beijing 102617, China

## Abstract

*Background*. Cancer is a significant disease burden in the world. Many studies showed that heavy metals or their compounds had connection with cancer. But the data conflicting about the relationship of manganese (Mn) to cancer are not enough. In this paper, the relationship was discussed between Mn concentrations in drinking water for rural residents and incidence and mortality caused by malignant tumors in Huai'an city. *Methods*. A total of 158 water samples from 28 villages of 14 towns were, respectively, collected during periods of high flow and low flow in 3 counties of Huai'an city, along Chinese Huai'he River. The samples of deep groundwater, shallow groundwater, and surface water were simultaneously collected in all selected villages. Mn concentrations in all water samples were determined by inductively coupled plasma-mass spectrometry (ICP-MS 7500a). The correlation analysis was used to study the relationship between the Mn concentration and cancer incidence and mortality. *Results*. Mn concentrations detectable rate was 100% in all water samples. The mean concentration was 452.32 *μ*g/L ± 507.76 *μ*g/L. There was significant difference between the high flow period and low flow period (*t* = −5.23, *P* < 0.05) and also among deep groundwater, shallow groundwater, and surface water (*F* = 5.02, *P* < 0.05). The ratio of superscale of Mn was 75.32%. There was significant difference of Mn level between samples in the high flow period and low flow period (*χ* ^2^ = 45.62, *P* < 0.05) and also among deep groundwater, shallow groundwater, and surface water (*χ* ^2^ = 10.66, *P* < 0.05). And also we found that, during the low flow period, Mn concentration has positive correlation with cancer incidence and mortality; for a 1 *μ*g/L increase in Mn concentration, there was a corresponding increase of 0.45/100000 new cancer cases and 0.35/100000 cancer deaths (*P* < 0.05). *Conclusions*. In Huai'an city, the mean concentration of Mn in drinking water was very high. Mn concentration correlated with cancer incidence and mortality.

## 1. Background

The global burden of cancer is increasing largely as a result of population aging and growth. Based on the GLOBOCAN 2008 estimates, about 12.7 million cancer cases and 7.6 million cancer deaths are estimated to have occurred in 2008 [1]. Cancer is also one of the leading causes of deaths in China; it remains a significant disease burden. The crude incidence in Chinese cancer registration areas was 285.91/10^5^, and the crude mortality was 180.54/10^5^ [2]. The complex pathogenesis of cancer is considered as the interaction between environment exposures and genetics with multiple factors and steps. It is reported that environmental factors are the most important factors for cancer, especially, chemical factors which up to 80%–90% [3]. Huai'an city lies downstream of Huaihe Downriver River and has a higher cancer incidence which has been taken into account [4, 5].

Manganese (Mn) is widely distributed as one of the most abundant elements in the earth's crust and also in many foodstuffs. Mn is an essential trace element with many biological functions but toxic at higher doses [6, 7]. In industry, the majority of Mn is used to make alloys and steel. Interest in environmental manganese has increased recently while Mn is replacing lead as an antiknock agent in gasoline globally in the form of methylcyclopentadienyl manganese tricarbonyl (MMT) [8, 9]. A number of metals and metal compounds have been classified by the International Agency for Research on Cancer (IARC) as known carcinogens, but Mn and its compounds have not been evaluated [10]. Many studies show that Mn has an important role in neurodegenerative diseases [11]. But the data conflicting about the relationship of manganese to cancer are not enough. In this paper, we mainly discussed the relationship between Mn concentrations in drinking water for rural residents and incidence and mortality caused by malignant tumors.

## 2. Materials and Methods

### 2.1. General Situation of Study Area

Huai'an city is located in the center of Jiangsu province across Huaihe River. It has 5.4 millionpeople and 70% of them are rural residents. It has four counties, four districts, and one economic development zone which contain in all 116 towns. Here it has a high cancer incidence especially digest system neoplasm. Currently, only Huai'an city and its urban district use central water supply, while the other towns use small central water supply in rural areas. Before quality improvement, drinking water for rural residents mainly came from well and hand pressure well or earlier from the ditch and pond.

### 2.2. Water Sample

We chose 5 towns from Jinhu County and Chuzhou county, respectively, and 4 towns from Xuyi County and chose 2 villages with a higher cancer incidence of every chosen town. Finally, there were 28 villages. 158 water samples from these villages were, respectively, collected in two water period: the high flow and low flow period, and three water sources: deep groundwater, shallow groundwater, and surface water. About 500 mL of water was bottled and HNO_3_ was added to keep pH < 2. Then the samples were transported to the lab and were stored under 4°C. The water samples were analyzed by ICP-MS 7500a.

### 2.3. Data Analysis

According to national standards for drinking water quality, Mn concentrations more than 100 *μ*g/L were defined as overproof in drinking water. We use SAS9.1 for data analysis. The data of incidence and mortality of population Cancer from 2008 to 2010 year come from Cancer Registration System of Huai'an city. The correlation analysis was used to study the relationship between the Mn concentration and cancer incidence and mortality.

## 3. Results

### 3.1. Incidence and Mortality of Cancer

Cancer incidence and mortality of Chuzhou county, Jinhu county, and Xuyi county was 216.54/10^5^, 282.47/10^5^, and 179.33/10^5^ and 157.01/10^5^, 192.20/10^5^, and 170.02/10^5^. There was a significant difference in cancer incidence and mortality among these three counties (*P* < 0.01). This study included 158 water samples from 28 villages of 14 towns in Chuzhou county, Jinhu county, and Xuyi county. All of them, 84 water samples were from the high flow period and 74 water samples from the low flow period. Detectable rate was 100%.

### 3.2. The Comparison of Mn Concentrations in Drinking Water

The min concentration of Mn in water samples was 0.02 *μ*g/L, the max was 4364.00 *μ*g/L, and the mean was 452.32 *μ*g/L ± 507.76 *μ*g/L. The mean concentration of Mn in Chuzhou county, Jinhu county, and Xuyi county was, respectively, 401.15 *μ*g/L ± 309.76 *μ*g/L, 574.46 *μ*g/L ± 701.60 *μ*g/L, and 350.17 *μ*g/L ± 352.26 *μ*g/L. There was no significant difference in the mean concentration among these three counties (*F* = 2.92, *P* > 0.05). The mean concentration of Mn in the high flow period and the low flow period was 265.47 *μ*g/L ± 388.83 *μ*g/L, and 664.41 *μ*g/L ± 544.82 *μ*g/L, and there was a significant difference between them (*t* = −5.34, *P* < 0.05). The mean concentration of Mn in deep groundwater, shallow groundwater, and surface water was 287.83 *μ*g/L ± 370.25 *μ*g/L, 573.64 *μ*g/L ± 672.47 *μ*g/L, and 506.33 *μ*g/L ± 362.47 *μ*g/L, and there was a significant difference among them (*F* = 5.02, *P* < 0.05). The concentration of Mn in deep groundwater and shallow groundwater was more than in surface water; the difference was significant (*P* < 0.05). During the high flow period, the mean concentration of Mn in three water sources was difference significantly with the mean concentration in deep groundwater being only 72.01 *μ*g/L ± 130.91 *μ*g/L while the other two sources being 437.85 *μ*g/L ± 439.79 *μ*g/L, 286.56 *μ*g/L ± 429.09 *μ*g/L (*F* = 7.19, *P* < 0.05) (Table 1).

### 3.3. The Comparison of Mn Concentrations in Drinking Water during Different Period

During high flow period, the mean concentration of Mn in deep groundwater, shallow groundwater, and surface water was 437.85 *μ*g/L ± 439.79 *μ*g/L, 286.56 *μ*g/L ± 429.09 *μ*g/L, and 72.01 *μ*g/L ± 130.91 *μ*g/L, and there was a significant difference among them (*F* = 7.19, *P* < 0.05). During high flow period, the mean concentration of Mn in deep groundwater, shallow groundwater, and surface water was 607.24 *μ*g/L ± 168.38 *μ*g/L, 871.35 *μ*g/L ± 753.13 *μ*g/L, and 503.65 *μ*g/L ± 406.83 *μ*g/L, and there was a significant difference among them (*F* = 3.50, *P* < 0.05). S-N-K showed that in high flow period Mn concentration in both shallow groundwater and surface water were higher than that in deep groundwater (*P* < 0.05). During high flow period, Mn concentration in shallow groundwater was higher than that in deep groundwater (*P* < 0.05) (Table 2).

### 3.4. The Comparison of Superscale Ratio of Mn in Drinking Water

Referring to Standards for Drinking Water Quality, the ratio of superscale of Mn in 158 water samples was 75.32%. In Chuzhou county, Jinhu county and Xuyi county, the ratio of superscale of Mn was 80.70%, 77.97%, and 64.29%, the difference was not significant (*χ* ^2^ = 3.86, *P* > 0.05). The ratio of superscale of Mn in the high flow period and the low flow period was 53.57% and 100%; the difference was significant (*χ* ^2^ = 45.62, *P* < 0.05). The ratio of superscale in surface water was 87.23% which was the highest one. And next one was in shallow groundwater which was 80.00%. The third one was in deep groundwater which was 60.71%. The difference among them was significant (*χ* ^2^ = 10.66, *P* < 0.05) (Table 3).

### 3.5. The Comparison of Superscale Ratio of Mn in Drinking Water in Different Period

Be worth mentioning, the ratio of superscale in all water sources during the low flow period was 100%. But in high flow period, the ratio of superscale of three water sources was different, and the ratio was 78.57%, 60.71%, and 21.43%, respectively. The difference among three water sources was significant (*χ* ^2^ = 19.24, *P* < 0.05) (Table 4).

### 3.6. The Correlation Analysis

The correlation analysis indicated that the Mn concentration has positive correlation with cancer incidence and mortality (*P* < 0.05). Regression analysis showed that, for 1 *μ*g/L increase in Mn concentration, there was a corresponding increase of 0.45/10^5^ cancer cases and 0.35/10^5^ cancer deaths (*P* < 0.05) (Tables 5 and 6).

## 4. Discussion

Our study showed that the mean concentration of Mn in drinking water in Huai'an city was much higher than the concentration; refer to standards for drinking water quality. The ratio of superscale is high of 75.32% and during the low flow period was 100%. We also found that the difference between it in the high flow period and low flow period was significant. For three water sources, the ratio of superscale of Mn in surface groundwater and shallow groundwater was higher than deep in water, while the same as the mean concentration. Excessive and prolonged exposure to Mn resulted from welding and mining, inhalation of combustion products from the antiknock agent in fuel (MMT) and highly concentrated Mn concentrations in ground/well water, and so on [7]. In our investigation, these three counties have great quantity industries, such as machinery manufacturing, alternative construction material, chemical industry, and oil industry. So we deduced that Mn may come from local industrial pollution.

Another important finding was that Mn concentration in drinking water in Huai'an city correlated with cancer incidence and mortality. In our city, for a 1 *μ*g/L increase in Mn concentration, there was a corresponding increase of 0.45/10^5^ cancer cases and 0.35/10^5^ cancer deaths. At first glance, this finding appears to be unbelievable. A large ecological study was carried out in North Carolina, and they also found manganese concentration in groundwater correlate with cancer mortality [12]. There are also some other studies showed the relationship between Mn and cancer. Mn is a component of the important antioxidant enzyme, manganese peroxide dismutase (MnSOD) which mitigates the oxidative damage from reactive oxygen species, one pathway thought to participate in carcinogenesis, such as lung cancer and breast cancer [13, 14]. An inverse relationship between breast cancer and hair manganese was documented [15, 16].

Though World Health Organization (WHO) found among the general population, the average estimated Mn intake from drinking water is substantially lower than the intake of Mn from the diet, typically 20 *μ*g/d [17], our findings point out thatmental pollution should be taken into account. Factors such as age, chemical species, dose, route of exposure, and dietary components influence both the absorption and bioavailability of manganese in human [18–21]. Thus the measures for mental pollution prevention and control should be of diversity.

Because data were analyzed at the population level and these same county-level factors may be related to individual adverse health behaviors such as cigarette smoking and obesity that contribute to cancer incidence and mortality, these ecological findings should be confirmed at the individual level or in animal models.

## 5. Conclusion

The mean concentration of Mn in drinking water in Huai'an city was much higher than the concentration according to the national standards for drinking water quality. And the ratio of superscale is very high. Mn concentration is carcinogenic to people, which correlates with cancer incidence and mortality. Measures for mental pollution prevention and control must be taken immediately.

## Figures and Tables

**Table 1:** The comparison of Mn concentrations in drinking water in Huai'an city.

Variable	*N*	Mean	Std.	*t*(*F*)	*P*
Counties					
Chuzhou county	57	401.15	309.76	2.92	0.06
Jinhu county	59	574.46	701.6		
Xuyi county	42	350.17	352.26		
Periods					
High flow period	84	265.47	388.83	−5.34	0.00
Low flow period	74	664.41	544.82		
Sources					
Surface water	47	506.33	362.88	5.02	0.01
Shallow groundwater	55	573.64	672.47		
Deep groundwater	56	287.83	370.25		
All	158	452.32	507.76		

**Table 2:** The comparison of Mn concentrations in drinking water during different periods.

Periods	Water sources	*N*	Mean	Std.	*F*	*P*
High flow period	Surface water	28	437.85	439.79	7.19	0.00
	Shallow groundwater	28	286.56	429.09		
	Deep groundwater	28	72.01	130.91		
	All	84	265.47	388.83		

Low flow period	Surface water	19	607.24	168.38	3.50	0.04
	Shallow groundwater	27	871.35	753.13		
	Deep groundwater	28	503.65	406.83		
	All	74	664.41	544.82		

**Table 3:** The comparison of superscale ratio of Mn in drinking water.

Variable	Superscale		Qualified		*χ* ^2^	*P*
	*N*	%	*N*	%		
Counties						
Chuzhou county	46	80.70	11	19.30	3.86	0.15
Jinhu county	46	77.97	13	22.03		
Xuyi county	27	64.29	15	35.71		
Periods						
High flow period	45	53.57	39	46.43	45.62	0.00
Low flow period	74	100.00	0	0.00		
Sources						
Surface water	41	87.23	6	12.77	10.66	0.01
Shallow groundwater	44	80.00	11	20.00		
Deep groundwater	34	60.71	22	39.29		
All	119	75.32	39	24.68		

**Table 4:** The Comparison of superscale ratio of Mn in drinking water in different periods.

Variable	Superscale		Qualified		*χ* ^2^	*P*
	*N*	%	*N*	%		
Sources						
Surface water	22	78.57	6	21.43	19.24	0.00
Shallow groundwater	17	60.71	11	39.29		
Deep groundwater	6	21.43	22	78.57		
All	45	53.57	39	46.43		

**Table 5:** The correlation analysis between the Mn concentration and cancer incidence rate and mortality rate.

Variable	Incidence		Mortality	
	*r*	*P*	*r*	*P*
Periods				
High flow period	−0.12	0.68	−0.22	0.45
Low flow period	0.71	0.01	0.67	0.01
Sources				
Surface water	−0.14	0.62	−0.21	0.47
Shallow groundwater	0.47	0.09	0.34	0.24
Deep groundwater	0.21	0.47	0.40	0.16
All	0.20	0.21	0.17	0.27

**Table 6:** Regression analysis of correlation between Mn concentration and cancer incidence rate and mortality rate during the low flow period.

Variable	Incidence			Mortality		
	*β*	S.E	*P*	*β*	S.E	*P*
Mn	0.45	0.13	0.005	0.35	0.11	0.008
Intercept	19.92	79.66	0.81	3.29	68.32	0.96
